# Chinese Verbal Fluency Deficiency in Temporal Lobe Epilepsy with and without Hippocampal Sclerosis: A Multiscale Study

**DOI:** 10.1523/JNEUROSCI.0558-24.2024

**Published:** 2024-07-25

**Authors:** Kangrun Wang, Fangfang Xie, Chaorong Liu, Ge Wang, Min Zhang, Jialinzi He, Langzi Tan, Haiyun Tang, Bo Xiao, Lily Wan, Lili Long

**Affiliations:** ^1^Department of Neurology, Xiangya Hospital, Central South University, Changsha 410008, China; ^2^Department of Neurosurgery, First Affiliated Hospital of Wenzhou Medical University, Wenzhou Medical University Wenzhou, Wenzhou 325000, China; ^3^Clinical Research Center for Epileptic disease of Hunan Province, Xiangya Hospital, Central South University, Changsha 410008, China; ^4^National Clinical Research Center for Geriatric Disorders, Xiangya Hospital, Central South University, Changsha 410008, China; ^5^Department of Radiology, Xiangya Hospital, Central South University, Changsha 410008, China; ^6^Department of Anatomy and Neurobiology, Central South University Xiangya Medical School, Changsha 410008, China

**Keywords:** Chinese, functional MRI, temporal lobe epilepsy, verbal fluency

## Abstract

To test a Chinese character version of the phonemic verbal fluency task in patients with temporal lobe epilepsy (TLE) and assess the verbal fluency deficiency pattern in TLE with and without hippocampal sclerosis, a cross-sectional study was conducted including 30 patients with TLE and hippocampal sclerosis (TLE-HS), 28 patients with TLE and without hippocampal sclerosis (TLE-NHS), and 29 demographically matched healthy controls (HC). Both sexes were enrolled. Participants finished a Chinese character verbal fluency (VFC) task during functional MRI. The activation/deactivation maps, functional connectivity, degree centrality, and community features of the left frontal and temporal regions were compared. A neural network classification model was applied to differentiate TLE-HS and TLE-NHS using functional statistics. The VFC scores were correlated with semantic fluency in HC while correlated with phonemic fluency in TLE-NHS. Activation and deactivation deficiency was observed in TLE-HS and TLE-NHS (*p *< 0.001, *k* ≥ 10). Functional connectivity, degree centrality, and community features of anterior inferior temporal gyri were impaired in TLE-HS and retained or even enhanced in TLE-NHS (*p *< 0.05, FDR-corrected). The functional connectivity was correlated with phonemic fluency (*p *< 0.05, FDR-corrected). The neural network classification reached an area under the curve of 0.90 in diagnosing hippocampal sclerosis. The VFC task is a Chinese phonemic verbal fluency task suitable for clinical application in TLE. During the VFC task, functional connectivity of phonemic circuits was impaired in TLE-HS and was enhanced in TLE-NHS, representing a compensative phonemic searching strategy applied by patients with TLE-NHS.

## Significance Statement

Verbal fluency tasks are essential assessments for patients with epilepsy. However, the testing and applying of Chinese phonemic verbal fluency is at an initial stage. We tested a Chinese character verbal fluency task in Chinese with temporal lobe epilepsy and depicted the functional alteration patterns in them. Our work provided a Chinese verbal fluency task suitable for clinical application. Our results highlighted the importance of the left anterior inferior frontal gyrus in Chinese and might have an impact on the surgical protocol of Chinese patients with left TLE and promote further development of rehabilitative and therapeutic approaches for verbal fluency decline.

## Introduction

Verbal fluency decline is a common comorbidity in temporal lobe epilepsy (TLE). A verbal fluency test is an essential presurgical assessment for evaluating the prognosis and postsurgical cognitive outcome. Verbal fluency task–based functional MRI (fMRI) provides direct insight into language network reorganization due to TLE and predicts postsurgical language outcomes (Foesleitner et al., 2020).

Chinese is the mother tongue of ∼20% of the world’s population and the world's largest population of patients with epilepsy. In China, there were approximately 10 million people with epilepsy in 2016, with 400,000 new cases and 10,000 lesion resective surgery cases annually (Y. Lin et al., 2021). Despite the demand, the testing and applying of Chinese phonemic verbal fluency is at an initial stage. In Indo-European languages, the term “phonemic verbal fluency” is normally equal to “letter verbal fluency” since phonological processing begins with phonemes and is evoked by initial letters (O’Seaghdha et al., 2010). However, linguistic differences of Chinese had hindered the direct revision of letter verbal fluency. The graphemes in Chinese are Chinese characters, which form a graphic syllabic logography system. The phonological processing in Chinese begins with syllables, corresponding to Chinese characters (O’Seaghdha et al., 2010). In the Mandarin Pinyin system, Chinese characters are irreversibly converted into 1,305 toned syllables. According to linguistic features and the Pinyin system of Chinese, two candidates had been proposed for Chinese (Mandarin) phonemic verbal fluency: consonants of Mandarin Pinyin [Ci et al., 2016; verbal fluency Pinyin (VFP)] and Chinese characters [Ci et al., 2016; Song et al., 2020; verbal fluency character (VFC)]. In the VFP task, participants are required to name Chinese characters whose corresponding Mandarin Pinyin syllables start with a given consonant (Ci et al., 2016). For example, if the given consonant is “d,” the characters generated could be “电” (electricity, Pinyin in Mandarin is “dian”), “点” (dot, “dian”), and “蛋” (egg, “dan”). In the VFC task, participants are required to name Chinese words that start with a given Chinese character. For example, if “电” is given, the words generated could be “电器” (electric appliance, “dian-qi”) and “电闪雷鸣” (lightning and thunder, “dian-shan-lei-ming”), whereas “点名” (roll call, “dian-ming”) is a wrong answer.

Both tasks triggered a similar but wider activation/deactivation effect compared with the letter verbal fluency task (Ci et al., 2016; Song et al., 2020), suggesting that they are eligible for Chinese phonemic verbal fluency tasks. VFP test imitates the letter verbal fluency task in Indo-European languages and would not involve Chinese characters. Hence, the VFP task could be considered as a “pure” phonemic verbal fluency task. However, the Mandarin Pinyin system is a pedagogical tool generally used in primary education but rarely used in everyday life (D. Lin et al., 2010). Hence, the VFP task is highly influenced by education level and deviates from the daily language environment. In contrast, the VFC task is closer to everyday situations and more standardized, providing a robust tool for routine or pre- and postsurgical assessment in Chinese patients with TLE.

Two types of verbal fluency are tested in TLE: phonemic verbal fluency and semantic verbal fluency. Verbal fluency processing is based on the dual stream model as phonemic verbal fluency relies mainly on the left frontal lobe and semantic verbal fluency also depends on the left posterior temporal lobe (Schmidt et al., 2019; Biesbroek et al., 2021). Lesion and fMRI studies had refined the anatomical map of verbal fluency. In 1,231 stroke cases, phonemic verbal fluency decline was attributed to lesions at the left anterior middle and inferior temporal gyrus (aMTG and aITG), while semantic verbal fluency decline was attributed to damages of the left temporo-occipital part of the middle and inferior temporal gyrus (toMTG and toITG; Biesbroek et al., 2021). Meanwhile, the triangularis part of the left inferior frontal gyri (left triIFG) and opercularis part of the left inferior frontal gyri (left operIFG) were correlated with semantic and phonemic verbal fluency, respectively (Costafreda et al., 2006; Ralph et al., 2017; Biesbroek et al., 2021). Different from Latin alphabets, a single Chinese character has its specific meaning. A previous study in neurotypical controls observed the involvement of both phonemic and semantic white matter circuits during the VFC task (Chang et al., 2020).

This study aimed to depict the functional characteristics revealed by the VFC task in healthy controls (HC), patients with TLE and hippocampal sclerosis (TLE-HS), and patients with TLE and without hippocampal sclerosis (TLE-NHS) and to explore the neural basis of verbal fluency decline in TLE. We applied three approaches to the left frontal and temporal regions: (1) activation/deactivation pattern, which reflects the involvement of phonemic or semantic regions; (2) functional connectivity and degree centralities (DC), which indicates the strength of phonemic or semantic circuits; and (3) affinity, which characterizes the functional consistency of a region of interest (ROI) and phonemic or semantic regions. We hypothesized that both phonemic and semantic circuits are evoked during the VFC task, while neuropsychological disorders, e.g., TLE, disrupt phonemic and semantic verbal fluency at different levels (Zalonis et al., 2017).

## Materials and Methods

### Subjects

From December 2018 to January 2021, 58 patients with TLE (male:female = 29:29) were sequentially recruited from Xiangya Hospital. Twenty-nine demographically matched healthy controls (male:female = 14:15) were recruited from the same social background. Since cerebral language function is lateralized, we restricted this study to right-handed participants. The inclusion criteria were as follows: (1) no history of neurological or psychiatric disease except for TLE, (2) 16–65 years old, (3) right-handed native Chinese, (4) fluent in Mandarin and not able to speak Cantonese, and (5) able to comprehend and complete our language paradigm.

The clinical semiology, electroencephalography, and neuroimages were evaluated by two experienced epileptologists (B.X. and L.L.) to diagnose and lateralize TLE (Fisher et al., 2014). The criteria for hippocampal sclerosis were as follows: (1) visually decreased hippocampal volume, increased temporal horn volume, gray–white matter boundary blurring, asymmetrical hippocampus, loss of internal structure, or increased T2 signal (Mo and Liu, 2019), evaluated by experienced neuroimagers (F.X. and H.T.), and (2) smaller than the 95% reference hippocampus volume, calculated automatically by Hipposeg (Winston et al., 2013). A blind rater (L.L.) reconciled the disagreement between the neuroimagers and automatic segmentation (Cook et al., 1992).

Written informed consent was obtained from all participants. The study was approved by the ethics committee of Xiangya Hospital of Central South University.

### Neuropsychological tests

All participants finished a battery that contained the following: (1) self-rating anxiety scale (SAS; Zung, 1971) and self-rating depression scale (SDS; Zung, 1965) for psychiatric comorbidity; (2) Montreal cognitive assessment (MoCA; Nasreddine et al., 2005) to assess the overall cognitive function; (3) verbal fluency semantic (VFS) test for semantic verbal fluency; (4) VFP (Ci et al., 2016) test for phonemic verbal fluency; and (5) VFC test to estimate participants’ performance during the scan.

### Magnetic resonance data acquisition

At the MRI center of Xiangya Hospital, MRI images were collected with a Siemens MAGNETOM Prisma 3.0T MR scanner and a standard head coil. The whole-brain structural images were obtained using a magnetization-prepared rapid acquisition with gradient echo sequence (field of view, 233 mm; repetition time, 2.11 s; echo time, 3.18 ms; flip angle, 9°; 320 × 320 matrix), and the whole-brain blood oxygenation level-dependent (BOLD) signal images were provided by a gradient echo-planar T2-weighted sequence (field of view, 225 mm; repetition time, 1 s; echo time, 37 ms; flip angle, 52°; 90 × 90 matrix).

### Language paradigm

During the fMRI scanning, participants were instructed to finish the covert VFC task (Ci et al., 2016). The task contained five blocks, with a 30 s rest module and a 30 s task module in each block. During the rest modules, participants were asked to look at the crosshair fixation on a white background and rest. One Chinese character would be shown on the white screen during each task module. Participants were required to covertly generate words that start with the given Chinese character. We selected five characters frequently used in daily life for task modules: “天” (sky, “tian”), “电” (electricity, “dian”), “文” (script, “wen”), “花” (flower, “hua”), and “水” (water, “shui”).

### fMRI images preprocessing

Imaging data were preprocessed with the Statistical Parametric Mapping 12 (SPM12, https://www.fil.ion.ucl.ac.uk/spm/). The preprocessing pipeline includes realignment, coregistration, segmentation, normalization to the Montreal Neurological Institute (MNI) space, and spatial smoothing (6 mm).

### Main effect analysis

Main effect analysis was conducted within cerebral regions with definite phonemic or semantic fluency assignment (left frontal and temporal cortex; Desikan et al., 2006). A two-level generalized linear model (GLM) was established with SPM12. At the individual level, we calculated the task-dependent contrast images for each participant. Lateralization indexes (LI) of contrast images in the frontal lobe were calculated for each participant with the LI-toolbox embedded in SPM12 (Wilke and Lidzba, 2007). LI is defined as left-dominant (LI > 0.2), right-dominant (LI < −0.2), and bilateral-dominant (−0.2 ≤ LI ≤ 0.2).

One sample *t* test was applied to individual images of HC to generate a group-level activation/deactivation map. One-way analysis of covariance (ANCOVA) and post hoc pairwise comparisons were used to estimate the difference between HC, TLE-HS, and TLE-NHS, with sex, age, education level, and MoCA as covariates. Clusters were considered significant under a threshold of *p *< 0.001, with an extent threshold of 10-voxel minimum cluster size (Wang et al., 2022).

### Functional statistics for ROIs

We used toolbox CONN v.20.b (Whitfield-Gabrieli and Nieto-Castanon, 2012) to calculate functional connectivity matrices. The brain was parcellated into 91 cortical regions, 15 subcortical regions, and 26 cerebellar regions according to the FSL Harvard-Oxford Atlas maximum likelihood atlas (Desikan et al., 2006) and automated anatomical labeling atlas (Tzourio-Mazoyer et al., 2002). Left triIFG, left operIFG, left aMTG, left aITG, left toMTG, and left toITG were selected as ROIs. Outlying scans were detected by the functional outlier detection tool. Head motion, outlying scans, the effect of modules, and BOLD signals inside white matter and cerebral spinal fluid were regressed out. BOLD signals within 0.009–0.10 Hz were retained to remove high- and low-frequency noises (Amiri et al., 2020). Pearson’s correlation coefficients were calculated between brain parcellations to generate a 132 × 132-weighted matrix for each subject. Pearson’s correlation coefficients between ROIs and other brain regions (6 × 132) were compared between groups with ANCOVA and post hoc pairwise comparison.

For ROIs with significant group differences in functional connectivity analysis, we further calculated their DC and affinity to the frontal or temporal cortex. The DC is the number of suprathreshold functional connections linked to a specific node. The 132 × 132-weighted matrix was binarized into unweighted matrix with connection density ranging from 5 to 40%, in steps of 1% (Caciagli et al., 2020). The areas under the curve (AUCs) of DCs were compared between groups.

Functional connectivity between regions in the left frontal and temporal cortex was extracted for community detection. We used a Louvain-like algorithm (Jeub et al., 2011–2019) to optimize a modularity quality function (Newman, 2006) developed for dividing networks into communities. Considering the stochastic nature of the method, we performed 100 optimizations for each participant. $A_{i}^{X}$, the affinity of region *i* to cortex × (left frontal or temporal), is defined as:

$$A_{i}^{X}=\frac{1}{n_{X}^{i}}\sum_{\;j\in X,j\neq i}P_{ij},$$

where $n_{X}^{i}$ is the number of regions in cortex *X* except for region *i* and $P_{ij}$ is the probability that region *i* and *j* are in the same community in 100 optimizations.

### Classification models

As a validation and compensation of the abovementioned methods, the classification model provides a combined effect of functional difference between TLE-HS and TLE-NHS. Verbal fluency scores and functional statistics with significant group difference were selected to differentiate TLE-HS and TLE-NHS. We used logistic regression based on forward stepwise regression of maximum likelihood estimation to select parameters with discriminative power (*p *< 0.05). Parameters were then used to train and test a single-layer feed-forward neural network classification model with the following settings: lambda initial = 0.0000005, sigma initial = 0.00005, interval center = 0, and interval offset = 0.5. We randomly divided samples into training (70%) and testing (30%) subsets and performed 100 optimizations.

### Statistical analysis

Statistical analysis was conducted by RStudio (https://www.rstudio.com/) and IBM SPSS Statistics 23 (https://www.ibm.com/products/spss-statistics). When comparing qualitative variables, we used the Kruskal–Wallis test, ANCOVA, Quade nonparametric ANCOVA and post hoc pairwise comparisons, or Mann–Whitney *U* test when applicable. To compare categorical variables between groups, the chi-square test or Fisher’s exact test was applied.

The language scores were standardized against those of HC for correlation analysis. Partial correlation between VFC scores and phonemic or semantic verbal fluency scores was calculated in groups to estimate phonemic and semantic contribution during the VFC task. Also, partial correlation coefficient was calculated between language scores and functional statistics with significant group difference. The age, sex, educational level, and MoCA were controlled as covariates of no interest when comparing neuropsychological scores and functional statistics and calculating the partial correlation coefficient.

The α level was set at *p *< 0.05 with appropriate FDR correction.

Data support this article is available on request.

## Results

### Demographic and clinical data

HS was diagnosed in 30 patients with TLE. No significant group difference was noted in sex, age, and education level. The two patient groups did not differ in clinical characteristics. Compared to HC, TLE-HS had worse performance in MoCA (*p *= 0.02, FDR-corrected), SAS (*p *= 0.01, FDR-corrected), SDS (*p *= 0.001, FDR-corrected), and VFP (*p *= 0.002, FDR-corrected). Compared to HC, TLE-NHS also had worse performance in MoCA (*p *= 0.03, FDR-corrected), SAS (*p *= 0.008, FDR-corrected), and SDS (*p *= 0.01, FDR-corrected). TLE-NHS outperformed TLE-HS in the VFP test (*p *= 0.03, FDR-corrected). In addition, patients presented a trend for worse VFS and VFC performance. The lateralization of TLE-HS was more atypical compared with HC (*p *= 0.002, FDR-corrected) and TLE-NHS (*p *= 0.002, FDR-corrected). There was atypical language dominance (bilateral or right-sided) in two HCs, nine patients with TLE-HS, and three patients with TLE-NHS. Details are provided in Table 1.

**Table 1.** Demographic and clinical data

	HC	TLE-HS	TLE-NHS	Statistic	*p*
*N*	29	30	28	-	-
Age, years, median (IQR)	26.0 (18.0)	28.5 (9.0)	30.0 (12.0)	1.36^a^	0.51
Sex, male/female	14/15	14/16	15/13	0.30^b^	0.86
Education, years, median (IQR)	12.0 (7.0)	10.5 (7.0)	12.0 (4.0)	3.20^a^	0.20
MoCA, median (IQR)	29.0 (3.0)	26.0 (5.0)	26.0 (6.0)	8.55^a^	0.03*
LI, median (IQR)	0.82 (0.22)	0.65 (0.58)	0.84 (0.24)	8.45^a^	0.003*
VFC, median (IQR)	24.0 (15.0)	17.5 (9.0)	18.5 (9.0)	3.18^c^	0.05
VFP, median (IQR)	36.0 (28.0)	20.0 (17.0)	27.0 (21.0)	7.56^c^	0.004*
VFS median (IQR)	45.0 (29.0)	35.0 (13.0)	39.0 (13.0)	2.92^c^	0.06
SAS, median (IQR)	38.0 (8.0)	45.5 (13.0)	46.5 (16.0)	6.40^c^	0.008*
SDS, mean (SD)	39.3 (10.5)	49.5 (9.6)	47.3 (10.0)	8.36^d^	0.003*
Duration, median (IQR)	-	9.5 (14.0)	10.0 (17.0)	0.41^e^	0.68
AOO, median (IQR)	-	17.0 (10.0)	17.5 (11.0)	0.36^e^	0.72
Laterality, left/right	-	13/17	12/16	0.00^b^	0.97
Febrile convulsion history	-	2 (6.7%)	1 (3.6%)	-^f^	1.00
FTBTCS history		21 (70.0%)	25 (89.3%)	3.28^b^	0.08
Number of ASMs				-^f^	0.22
1	-	16	20		
2	-	13	8		
3	-	1	0		
Frequency	-			-^f^	0.68
Yearly	-	7	8		
Monthly	-	13	8		
Weekly	-	6	6		
Daily		4	6		

AOO, age of onset; ASMs, antiseizure medications; FTBTCS, focal to bilateral tonic–clonic seizures; HC, healthy controls; IQR, interquartile range; LI, lateralization index; MoCA, Montreal cognitive assessment; SAS, self-rating anxiety scale; SD, standard deviation; SDS, self-rating depression scale; TLE-HS, patients with temporal lobe epilepsy and hippocampal sclerosis; TLE-NHS, patients with temporal lobe epilepsy and without hippocampal sclerosis; VFC, verbal fluency character test; VFP, verbal fluency Pinyin test; VFS, semantic verbal fluency test.

a *H* values of Kruskal–Wallis test.

b *χ*^2^ value of Chi-squared test.

c *F* value of Quade nonparametric analysis of covariates.

d *F* value of analysis of covariates.

e *Z* value of Mann–Whitney *U* test.

f Fisher exact test.

* FDR-corrected.

### fMRI main effect

**Figure 1.**
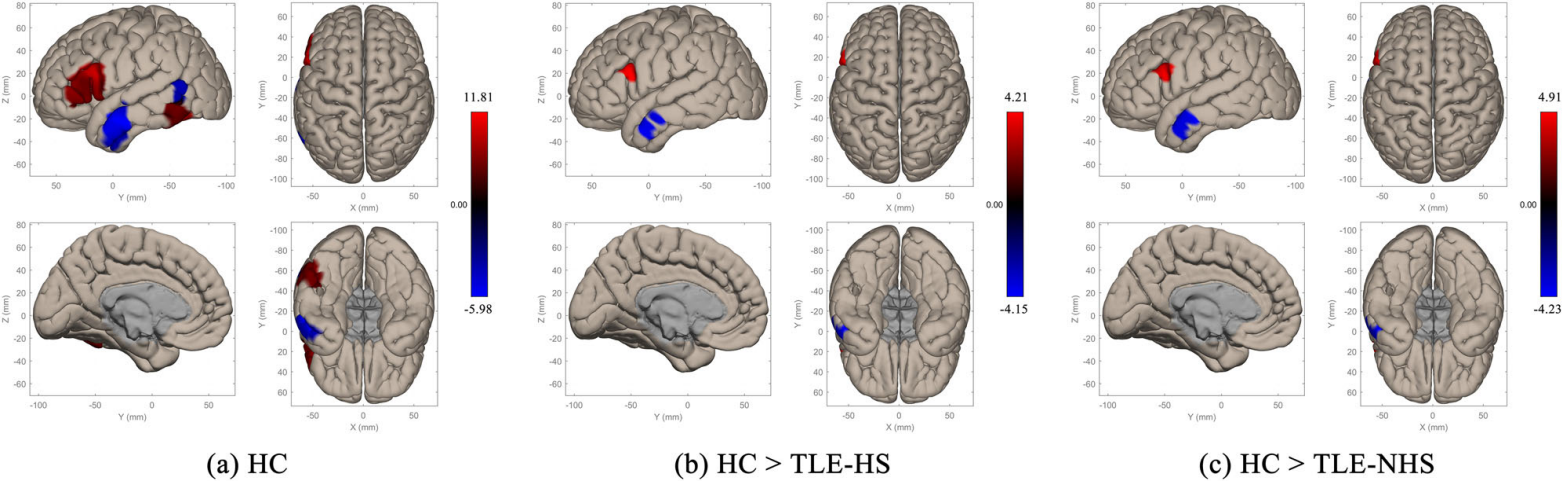
Activation/deactivation maps of the verbal fluency character task. ***a***, activation/deactivation map of HC; ***b***, group comparison between HC and TLE-HS; ***c***, group comparison between HC and TLE-NHS. Clusters are shown under the threshold: *p* < 0.001, *k* ≥ 10. HC, healthy controls; TLE-HS, patients with temporal lobe epilepsy and hippocampal sclerosis; TLE-NHS, patients with temporal lobe epilepsy and without hippocampal sclerosis.

VFC task deactivated the most of left temporal cortex and part of the left superior frontal gyri and activated most of the left frontal cortex, left toITG, and inferior part of the left toMTG (Fig. 1*a*). Compared to HC, the activation in the left triIFG and left operIFG was decreased, and the deactivation in the left aMTG and left aITG was decreased (*p *< 0.001, *k* ≥ 10) in TLE-HS (Fig. 1*b*) and TLE-NHS (Fig. 1*c*).

### Functional statistics for ROIs

**Figure 2.**
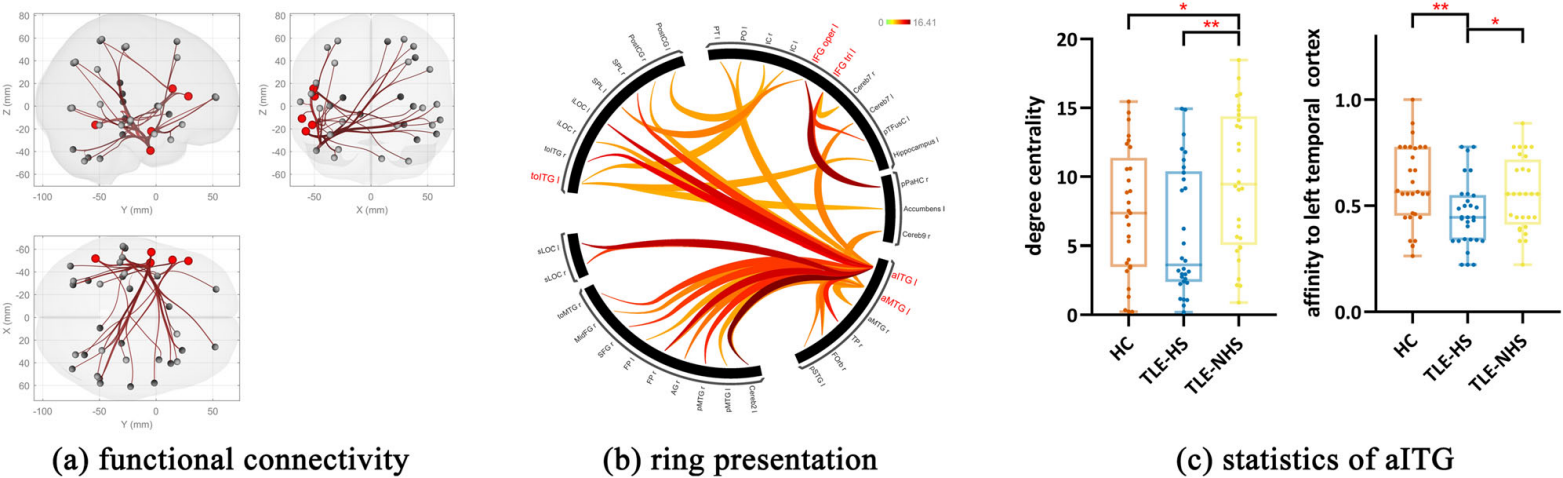
Functional network statistics for ROIs. ***a***, Glass. ***b***, Ring figures for functional connectivity under *p* < 0.05, FDR-corrected; ***c***, degree centrality and affinity to left temporal cortex of left aITG. **p < 0.005, FDR-corrected; *p < 0.05, FDR-corrected; l, left; r, right; AG, angular gyri; aMTG, anterior middle temporal gyri; aITG, anterior inferior temporal gyri; Cereb2, cerebellum crus II; Cereb7, cerebellum lobule VIIb; FOrb, frontal orbital cortex; FP, frontal pole; HC, healthy controls; IC, insular cortex; IFG oper, opercularis part of inferior frontal gyri; IFG tri, triangularis part of inferior frontal gyri; iLOC, inferior lateral occipital cortex; MFG, middle frontal gyrus; MidFG, middle frontal gyri; pMTG, posterior middle temporal gyri; PO, parietal operculum cortex; PostCG, postcentral gyri; pPaHC, posterior parahippocampal cortex; pSTG, posterior superior temporal gyri; PT, planum temporale; pTFusC, posterior temporal fusiform cortex; SFG, superior frontal gyri; sLOC, superior lateral occipital cortex; SPL, superior parietal lobule; TLE-HS, patients with temporal lobe epilepsy and hippocampal sclerosis; TLE-NHS, patients with temporal lobe epilepsy and without hippocampal sclerosis; toITG, temporo-occipital part of inferior temporal gyri; toMTG, temporo-occipital part of middle temporal gyri; TP, temporal pole.

During the task, functional connectivity seeding from the left triIFG, operIFG, aMTG, aITG, and toITG was different between the three groups while the majority of different functional connectivity was seeding from the left aITG (*p *< 0.05, FDR-corrected; Fig. 2*a,b*). Post hoc analysis revealed that TLE-HS had diminished functional connectivity compared with HC and TLE-NHS.

The DC and affinity of left triIFG, operIFG, aMTG, aITG, and toITG were compared between three groups (Fig. 2*c*). The left aITG in TLE-NHS had higher DC compared with HC (*p *= 0.04, FDR-corrected) and TLE-HS (*p *= 0.003, FDR-corrected). Meanwhile, the left aITG in TLE-HS presented lower affinity to the left temporal lobe compared with HC (*p *= 0.004, FDR-corrected) and TLE-NHS (*p *= 0.04, FDR-corrected).

### Neural network classification model

**Figure 3.**
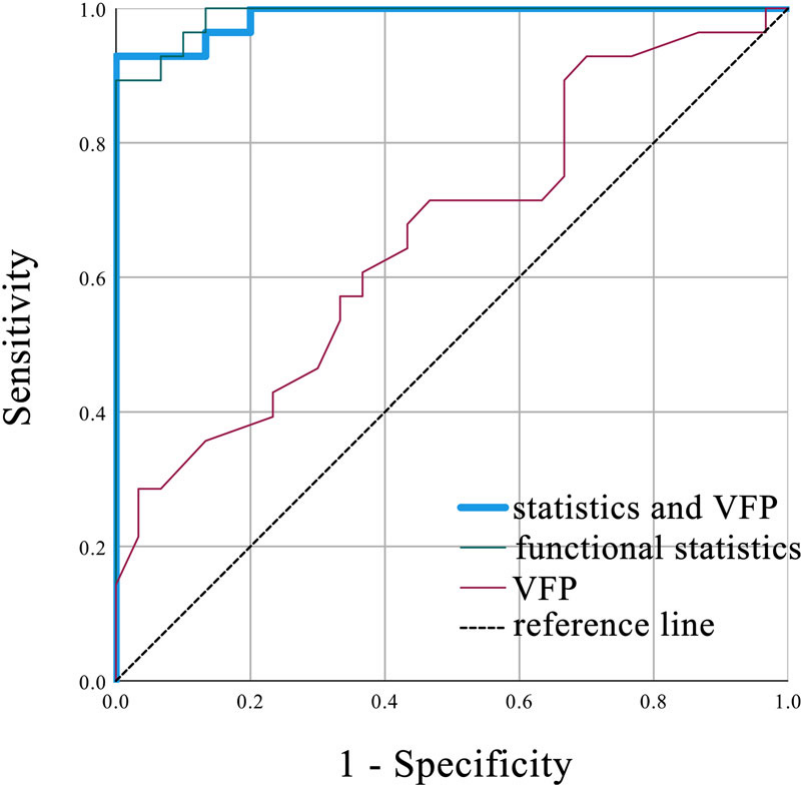
Logistic regression models differentiating TLE-HS and TLE-NHS. TLE-HS, patients with temporal lobe epilepsy and hippocampal sclerosis; TLE-NHS, patients with temporal lobe epilepsy and without hippocampal sclerosis; VFP, verbal fluency Pinyin.

VFP scores and functional connectivity had significant power for differentiating TLE-HS and TLE-NHS. The logistic regression model built with VFP scores had an AUC = 0.66 (95% CI = 0.525–0.805). The logistic regression model built with functional statistics reached an AUC = 0.99 (95% CI = 0.972–1.000; Fig. 3). The combination of statistics reached an AUC = 0.90 (95% CI = 0.833–0.974) in 100 repetitions of the neural network classification model.

### Correlation analysis

**Figure 4.**
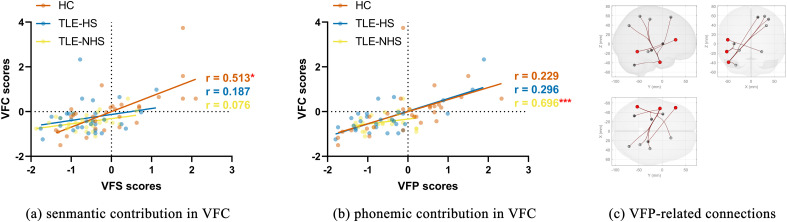
Correlation analysis. ***a***, Correlation between VFS and VFC scores; ***b***, correlation between VFP and VFC scores; ***c***, functional connectivity that correlated with VFP scores (*p* < 0.05, FDR-corrected). ***p < 0.001, FDR-corrected; *p < 0.05, FDR-corrected; HC, healthy controls; TLE-HS, patients with temporal lobe epilepsy and hippocampal sclerosis; TLE-NHS, patients with temporal lobe epilepsy and without hippocampal sclerosis; VFC, verbal fluency character; VFP, verbal fluency Pinyin; VFS, verbal fluency semantic.

VFC scores were correlated with semantic verbal fluency in HC (*p *= 0.03, FDR-corrected) and were correlated with phonemic verbal fluency in TLE-NHS (*p *< 0.001, FDR-corrected). In TLE-HS, VFC scores were not correlated with VFP or VFS scores (Fig. 4*a,b*).

Stronger (*p *< 0.05, FDR-corrected) functional connectivity was related to higher VFP scores (Fig. 4*c*). The functional connectivity between left toITG and left insular cortex was also correlated with VFS scores under a less robust threshold (*p *< 0.01, uncorrected).

### Sensitivity analysis

Although the impact of age, sex, education level, and MoCA scores was regressed out, several confounders could still bias the result. Hence, we conducted an extensive correlation analysis between the functional statistics and clinical features including (1) age of onset, (2) disease duration, (3) seizure frequency, (4) number of antiseizure medications, and (5) history of focal to bilateral tonic–clonic seizures. Clinical factors were also used as covariates of no interest in the TLE-HS versus TLE-NH comparison. In addition, we addressed the effect of (6) laterality of seizure focus by separate analysis and the effect of topiramate and carbamazepine by subgroup analysis.

In short, clinical features were not correlated with functional statistics and did not bias the group comparison. The group difference in left TLE was less prominent. The post hoc functional network statistics analysis in HC, right TLE-HS, and right TLE-NHS virtually replicated the whole-group analysis. Topiramate and carbamazepine did not affect the activation pattern of patients but reduced activation and deactivation under an exploratory threshold.

## Discussion

In this study, we tested the VFC task in patients with TLE. TLE-HS showed impaired functional connectivity seeding from the frontal and temporal cortex, while TLE-NHS had enhanced phonemic functional connectivity. We hypothesized that while HC preferred a semantic searching strategy for the VFC task, patients with TLE-NHS used a phonemic searching strategy to compensate for the impaired semantic verbal fluency ability.

Patients with TLE often suffer from verbal fluency deficiency. Verbal tasks locate the language network disruption in TLE and were used for individualized surgical (Bonelli et al., 2012; Osipowicz et al., 2016; You et al., 2019) or pharmacological (Wang et al., 2022) treatment outcome prediction. Here, we tested the verbal fluency character task in HC and patients with TLE. The VFC task evoked widespread activation and deactivation across the left frontal and temporal gyrus. Notably, regions specifically involved in semantic verbal fluency, including the left toMTG and toITG, were activated. Previous studies suggested that the left toMTG linked the semantic control system, DMN, and multiple-demand network, regulating semantic retrieval and top-down constraint during semantic tasks (Noonan et al., 2013; Davey et al., 2016; Ralph et al., 2017). Interestingly, another independent study using the VFC task also observed a correlation between VFC performance and semantic-related white matter tracts (Chang et al., 2020). Several points might account for the semantic involvement during the Chinese character phonemic task. First, different from a single letter, a single Chinese character has its specific meaning. Hence, semantic and phonemic searching strategies both fit the VFC task. For instance, “水井” (well, “shui-jing”), “水管” (water pipe, “shui-guan”), and “水瓶” (bottle, “shui-ping”) are all semantically and phonologically related with “水” (water, “shui”). Second, lexical orthographic knowledge was involved in the phonemic processing of Chinese characters (Wu et al., 2012), possibly because of the homophone density effect (Chen et al., 2016; Chao et al., 2021). In Chinese, over 8,000 frequently used characters share 1,305 toned syllables, resulting in a more prominent homophone density effect. Third, the activation in left toITG was related to higher cognitive demand (Davey et al., 2016). Fewer words were generated in the VFC task compared with letter verbal fluency tasks (8.5 words/min vs over 20 words/min), indicating that the cognitive burden for the VFC task was higher than that for letter verbal fluency tasks.

To further investigate the Chinese verbal fluency deficiency in TLE, we explored the verbal fluency scores and functional statistics of ROIs and their correlation. The semantic verbal fluency was impaired at roughly the same level in TLE-HS and TLE-NHS, while TLE-NHS had better phonemic fluency than TLE-HS. Consistent with impaired phonemic and semantic verbal fluency ability, the ROI-level statistics of TLE-HS were disturbed, mainly at left aITG. The left aITG was less integrated into the left temporal cortex and the whole cerebral network in TLE-HS compared with HC and TLE-NHS. In contrast, the functional connectivity of left aITG was conserved, even enhanced in TLE-NHS. Graph theoretical analysis revealed that the importance of left aITG in the cerebral functional network was increased in TLE-NHS compared with HC. Meanwhile, the community feature of aITG in TLE-NHS remained similar to that in HC. Stroke lesions at the left aITG led to phonemic verbal fluency deficiency, but not semantic fluency decline (Biesbroek et al., 2021). Notably, we observed that stronger functional connectivity of left aITG was correlated with better phonemic verbal fluency performance, indicating that the aITG was involved in phonemic verbal fluency circuits. In addition, VFC scores were correlated with semantic ability in healthy Chinese participants, indicating that semantic searching strategy is an auxiliary or even preferred strategy for VFC in HC. In TLE-NHS, VFC performance correlated with phonemic ability. Hence, we hypothesized that during the VFC task, TLE-NHS relied more on phonemic searching strategy as compensation for semantic deficiency. Meanwhile, severe phonemic decline disabled the compensative phonemic circuit in TLE-HS. The neural network classification model demonstrated that the phonemic functional connectivity during the VFC task was distinguishable between patients with and without HS. The classification model also indicated that the inclusion of fMRI features could improve the performance of the hippocampal sclerosis diagnosis model. Subgroup analysis for left and right TLE emphasized the effect of focus laterality on the function of aITG. As all of our patients are right-handed, seizure attacks originating from the left hemisphere might have impeded the functional enhancement of aITG.

Verbal fluency task–based scales and fMRI capture the language impairment in patients with neuropsychiatric disorders. By mapping individualized language network and impairment pattern, verbal fluency task–based fMRI becoming a handy tool for predicting outcomes of surgery (Bonelli et al., 2012; Osipowicz et al., 2016; You et al., 2019) and ASM treatment (Wang et al., 2022). Our work validated the VFC task as a verbal fluency task in the Chinese population. In addition, our results highlighted the importance of the left aITG in Chinese. This under-appreciated region of Chinese verbal fluency is one of the target loci of standard anterior temporal lobectomy for left drug-resistant TLE (Kohlhase et al., 2021). In 2018, in the United States, 96% of epilepsy programs applied task–based fMRI for presurgical evaluation, and 44% of programs used language fMRI to localize language areas (Benjamin et al., 2018). The language-based fMRI LI was validated as the strongest predictor for postsurgery naming outcome in a recent large multicenter cohort study (Gross et al., 2022). By preserving personalized activation areas evoked by language task–based fMRI, neurosurgeons would preserve the naming ability of patients with TLE (You et al., 2019). Personalized function area mapping had also been applied in the rehabilitation of trauma (Ren et al., 2022). Our work could be the base for formulating personalized treatment protocol for Chinese patients with TLE help to determine surgical protocol which avoid excessive destruction of individualized cognitive-required regions and promote further development of rehabilitative and therapeutic approaches for verbal fluency decline.

This study has limitations. First, HS was diagnosed without histological evidence. To improve accuracy, we adopted clinically empirical features which had a 97.9% accuracy (Mo and Liu, 2019). Visual evaluation was conducted by two experienced neuroimagers who specialized in epilepsy. Automatic segmentation was also applied to improve performance. Second, there were only limited and heterogenous reports regarding the neural basis of verbal fluency in Chinese. Hence, the interpretation of our findings was based on research on verbal fluency in alphabetic languages and reports of phonemic and semantic processing in Chinese. We adopted convergent and consistent evidence to choose the ROIs. Still, a large sample study in healthy volunteers would promote the understanding and clinical application of verbal fluency tasks in Chinese. Third, lesion lateralization would affect the language performance and functional pattern in TLE. We tested the lateral effect in the post hoc subgroup analysis. Patients with right TLE presented verbal fluency deficiency identical to that in the whole group. Meanwhile, the pattern was less prominent in patients with left TLE. However, the logistic regression model differentiated left TLE-HS and left TLE-NHS with an AUC = 1.00, indicating significant functional pattern difference between the two groups. A less significant group difference could be attributed to a smaller sample size. Alternatively, the left lesion might have led to severe functional impairment of the left temporal cortex and impeded the functional reorganization in the left TLE. Fourth, antiseizure medications could have influenced the BOLD signals. Although we tested the effect of a number of antiseizure medications and the effect of activation-related ASMs in the sensitivity analysis, we are unable to address the effect of heterogenous kinds and combinations of antiseizure medications. Last, Berkson's bias shall not be ignored since our participants were recruited from Xiangya Hospital, the highest-level comprehensive epilepsy center in the antiepileptic medical facility network of China.

In conclusion, the VFC task is a verbal fluency task suitable for clinical application in Chinese patients with TLE because it is close to the situation in everyday life and it evokes a wide effect in the temporal lobe. During the VFC task, phonemic functional connectivity was impaired in TLE-HS and was enhanced in TLE-NHS, potentially representing a compensative phonemic searching strategy applied by TLE-NHS. The VFC task and Chinese verbal fluency deficiency pattern are promising tools for exploring hippocampal abnormality as they were distinguishable between patients with and without HS.
